# Who children spend time with after school: associations with objectively recorded indoor and outdoor physical activity

**DOI:** 10.1186/1479-5868-11-45

**Published:** 2014-03-30

**Authors:** Matthew Pearce, Angie S Page, Tom P Griffin, Ashley R Cooper

**Affiliations:** 1 The Institute for Sport, Physical Education and Health Sciences, The Moray House School of Education, The University of Edinburgh, St Leonard's Land, Holyrood Road, Edinburgh EH8 8AQ, Scotland; 2 Centre for Exercise, Nutrition and Health Sciences, School for Policy Studies, University of Bristol, 8 Priory Road, Bristol BS8 1TZ, UK

**Keywords:** Physical activity, Children, Context, Accelerometer, Global Positioning System (GPS), Diary

## Abstract

**Background:**

Understanding how the determinants of behaviour vary by context may support the design of interventions aiming to increase physical activity. Such factors include independent mobility, time outdoors and the availability of other children. At present little is known about who children spend their time with after school, how this relates to time spent indoors or outdoors and activity in these locations. This study aimed to quantify who children spend their time with when indoors or outdoors and associations with moderate to vigorous physical activity (MVPA).

**Methods:**

Participants were 427 children aged 10–11 from Bristol, UK. Physical activity was recorded using an accelerometer (Actigraph GT1M) and matched to Global Positioning System receiver (Garmin Foretrex 201) data to differentiate indoor and outdoor location. Children self-reported who they spent time with after school until bed-time using a diary. Each 10 second epoch was coded as indoors or outdoors and for ‘who with’ (alone, friend, brother/sister, mum/dad, other grown-up) creating 10 possible physical activity contexts. Time spent and MVPA were summarised for each context. Associations between time spent in the different contexts and MVPA were examined using multiple linear regression adjusting for daylight, age, deprivation and standardised body mass index.

**Results:**

During the after school period, children were most often with their mum/dad or alone, especially when indoors. When outdoors more time was spent with friends (girls: 32.1%; boys: 28.6%) than other people or alone. Regression analyses suggested hours outdoors with friends were positively associated with minutes of MVPA for girls (beta-coefficient [95% CI]: 17.4 [4.47, 30.24]) and boys (17.53 [2.76, 32.31]). Being outdoors with brother/sister was associated with MVPA for girls (21.2 [14.17, 28.25]) but not boys. Weaker associations were observed for time indoors with friends (girls: 4.61 [1.37, 7.85]; boys: (7.42 [2.99, 11.85]) and other adults (girls: 5.33 [2.95, 7.71]; boys: (4.44 [1.98, 6.90]). Time spent alone was not associated with MVPA regardless of gender or indoor/outdoor location.

**Conclusions:**

Time spent outdoors with other children is an important source of MVPA after school. Interventions to increase physical activity may benefit from fostering friendship groups and limiting the time children spend alone.

## Background

Physical activity during childhood confers health benefits throughout the lifespan [1,2]. Children aged 5–18 are recommended to engage in at least one hour of moderate-to-vigorous physical activity (MVPA) per day [3], but the majority of children in the UK do not meet this target [4,5]. Consequently the development and implementation of policies and programmes to change this behaviour is a major public health priority. The development of such strategies relies upon an understanding of the factors influencing physical activity [6]. Research investigating the correlates and determinants of physical activity can help identify target groups in need of intervention, and highlight mediating variables which could be manipulated to change behaviour [7]. However, this process is complex because physical activity is not a single action but a class of varied behaviours [8], and different types of physical activity have been demonstrated to have different determinants [9-11]. Consistent with an ecological approach to understanding health behaviours [12], it is also important to consider the environmental context (i.e. location, time period, other participants) in which physical activity occurs [9]. Describing the value of different environmental and social settings for physical activity could inform context-specific interventions [13]. One contextual characteristic which could influence children’s physical activity is who children spend their leisure time with. Leisure time is a key source of children’s physical activity, especially during the ‘critical hours’ immediately after school [14]. After school leisure time may be spent alone, with brother/sister, with friends, with mum/dad or other grown-ups. It is plausible that who children spend their time with influences the duration, intensity and types of physical activity they engage in [15]. However, little is known about who children spend their time with after school, or how this is associated with their level of MVPA.

It is well established that the time children spend outdoors is more actively spent than time spent indoors [16,17]. At present it is unclear whether this time is spent alone, supervised by adults, or with other children. Time spent unsupervised by adults is thought to contribute significantly to children’s daily physical activity [18], while freedom from adult rules and structure is an important feature of active free play [19]. In addition, child directed play has the potential to provide unique emotional, social and cognitive benefits [20]. However, it is suggested that children’s, and in particular girls’ independent physical activity is increasingly limited due to parental concerns about safety [21-24]. Since independent mobility is consistently associated with children’s physical activity [25-27], it is important to quantify how much unsupervised outdoor time children are afforded. Time spent with friends and siblings and the availability of other children to play with have been reported to be key influences on children’s participation in unstructured outdoor physical activity [18,28-30]. These relationships may be of particular importance for children at the transition from primary to secondary school, as it is at approximately this age that independence from adults starts to develop [28,31].

To date, mostly qualitative and self or proxy report data have been used to characterise children’s indoor and outdoor after school leisure time physical activity. Objective information from Global Positioning System (GPS) receivers and accelerometers can more accurately quantify time spent indoors and outdoors in relation to physical activity [17]. Combining this with diary data reporting who children spend their time with provides a unique data set to describe a potentially important context for how physical activity may be modified. Consequently, the aim of this study is to use combined diary, GPS and accelerometer data to investigate who children spend their indoor and outdoor time with, and how this relates to MVPA after school. It is hypothesised that children’s MVPA accrual will vary by context, and that time spent outdoors with friends or siblings will exhibit stronger positive associations with MVPA than time spent in other contexts.

## Methods

This study used baseline data from the PEACH (Personal and Environmental Associations with Children's Health) project. Between September 2006 and July 2008 the project recruited 1,307 year six (age 10-11 yrs) children from 23 state primary schools in Bristol, UK. The methods of the PEACH project have been described fully elsewhere [25]. Written informed consent was obtained from a parent/guardian of all children who took part in the study. The study was carried out in accordance with the Declaration of Helsinki and ethical approval was provided by University of Bristol Ethics Committee.

### Physical activity

Physical activity intensity was summarised at ten second epochs using an accelerometer (GT1M; ActiGraph LLC, FL, USA). Participants were asked to wear the accelerometer on a waist belt for seven continuous days. The method of Troiano, Berrigan, Dodd et al. was used to identify accelerometer non-wear time: periods of 60 minutes (or more) of zero values were discarded allowing for up to two minutes of non-zeros per hour [32]. This criterion was used in preference to shorter non-wear definitions (e.g. 10 or 20 minutes) which can result in unnecessary removal of data and underestimation of sedentary time in some subgroups, for example those who are overweight [33]. For inclusion in analyses participants were required to have recorded at least three hours of after school accelerometer data on at least one weekday.

### Indoor/outdoor location

Positional data were recorded every ten seconds using a GPS receiver (GPS; Garmin Foretrex 201) [34]. Participants wore the GPS receiver between the end of school and bedtime on four consecutive school days. Participants were trained to turn the GPS receiver on at the end of school and off at bedtime. Research staff charged the units on day three of use due to limited battery life. Days with no GPS data were removed from the dataset.

### Diary data

Participants were asked to complete a one day recall diary for three school days. This diary was based on previous work [35,36]. The children were asked to record the start and end time of after school activities starting with the first thing they did after leaving school. In addition to the start and end time, participants were asked to select who they were with for each activity from five options: on my own, with friend, with brother/sister, with mum or dad, with another grown up. To maximise the quality of the diary provided by the children, an annotated example was provided and explained verbally by the researcher to small groups of participants (<10). Participants were incentivised to complete diaries via vouchers provided for completion of all measures and personal prompts were provided by researchers and teachers to remember to complete diaries. Periods with no diary record were quantified and children who did not provide diary data on at least one day were excluded from analyses.

### Confounding variables

Height (m) and weight (kg) were measured using a stadiometer (SECA) and digital scales (indoor clothing, shoes removed). Body mass index (BMI) was calculated (body mass in kg divided by height in metres squared), and BMI standard deviation score (BMISDS) was derived from standard tables [37]. Age, sex and post-code were confirmed by the Local Education Authority. Minutes of daylight from 15:00 until sunset for the day of measurement were determined using standard tables [38]. The UK Index of Multiple Deprivation (IMD) 2007 score was defined using full home postcode.

### Data processing

Ten second epoch accelerometer and GPS data were matched using date and time stamps for the period between 15:00 and 22:00 on weekdays using STATA (version 12.0, College Station, TX) as previously described [17]. Ten second epochs with accelerometer activity counts exceeding 383 (2296 counts per minute/6) were coded as MVPA [39,40]. The GPS receiver used in this study does not record positional data when inside a building. Consequently each epoch of accelerometer data with no corresponding GPS record was defined as indoors, while GPS matched accelerometer data were defined as outdoors. The delay between exiting a building and GPS signal acquisition can be as much as 45 seconds likely resulting in underestimation of time outdoors [17,41]. Any GPS point with a speed of greater than 15 kph was excluded as this was likely to represent an aberrant signal (e.g. reflection from a building) or motorised transportation [42].

Epochs with GPS and accelerometry data but no matching diary data entries were removed from the analyses. Participants who did not provide combined accelerometer, GPS and diary data on at least one day were excluded from analyses. In addition, some children provided diary entries with overlapping times and these were also excluded (<1% of total). Total minutes spent and minutes of MVPA were summed according to who children were with and whether they were indoors/outdoors. For example all epochs classified as ‘indoors’ and ‘with mum or dad’ were summed to give the time spent indoors with mum/dad, and the MVPA recorded during that time. This resulted in ten (indoors/outdoors: on own, with friend, with brother/sister, with mum/dad, with other grown-up) distinct contexts of after school physical activity.

### Data analyses

Descriptive statistics (mean, standard deviation and percentage) of total time and time in MVPA were calculated by sex, social company and location (indoors/outdoors). Multiple linear regression models were used to assess the contribution of time in each context to the total minutes of after school MVPA. This was expressed as the mean increase in minutes of MVPA for each hour spent in that context after adjusting for time spent in all the other contexts. In addition models were adjusted for potential *a priori* confounders (age, BMISDS, IMD, daylight hours). Due to well-established gender differences in daily physical activity, data for girls and boys were analysed separately [43]. Visual inspection of standardised residuals against predicted scores indicated some heteroskedacity and so robust (Huber-White) standard errors are reported. All analyses were conducted using Stata/SE (version 12.0, College Station, TX).

## Results

The sample consisted of 230 girls and 197 boys with mean age 10.7 (SD = 0.5) years and BMI 18.3 (SD = 3.2) kg/m^2^ who provided combined GPS, accelerometer and diary data on at least one measurement day. Overall, girls recorded 21.7 (SD = 12.3) minutes of MVPA (including data recorded both indoors and outdoors) during the after school period while boys recorded 25.0 (SD = 13.4) minutes. The GPS data estimated that girls spent 21.0 (SD = 27.7) minutes outdoors after school while boys were outdoors for 20.3 (SD = 27.4) minutes during the same period. Matched accelerometer and GPS data suggested that girls recorded 4.3 (SD = 6.4) minutes or 19.8% of total after school MVPA outdoors, while for boys this value was 4.6 (SD = 7.1) minutes or 18.4%. Girls provided a mean of 155.6 (SD = 71.9) minutes of after school diary information, and this was time-matched to accelerometer data which included on average 13.4 (SD = 9.3) minutes of MVPA. Boys provided a mean of 160.1 (SD = 74.5) minutes of after school diary information, and this was time matched to accelerometer data which included on average 15.6 (SD = 11.1) minutes of MPVA. Of all the valid after school accelerometer data, 40.5% of girls’ and 38.3% of boys’ accelerometer epochs could not be time matched to diary records because no diary entries had been recorded by the children during these periods. Consequently, 37.0% of girls’ and 36.0% of boys’ accelerometer recorded MVPA was not described by the participants in their diary. Table 1 reports the proportion of accelerometer epochs that were matched to GPS data (subsequently labelled outdoors), and the proportion of accelerometer epochs matched to a diary record, by hour.

**Table 1:** Proportion of accelerometer epochs matched to GPS and diary records by hour

**Hour**	**Total accelerometer epochs**	**Matched to GPS record**		**Matched to diary record**	
		**Total**	**%**	**Total**	**%**
15:00–15:59	331189	35420	10.7	133588	40.3
16:00–16:59	322970	34014	10.5	247820	76.7
17:00–17:59	323090	23290	7.2	233386	72.2
18:00–18:59	316641	26200	8.3	207956	65.7
19:00–19:59	270367	14106	5.2	163343	60.4
20:00–20:59	193725	7272	3.8	96611	49.9
21:00–21:59	84595	1687	2.0	31200	36.9

GPS: Global positioning system.

### Total time spent and MVPA

Using the available combined accelerometer, diary and GPS data, Table 2 (girls) and Table 3 (boys) summarise the time spent and MVPA recorded according to who children were with (from diary data) and whether they were indoors or outdoors after school (from GPS data). Both girls (28.9%) and boys (28.3%) recorded more time with their mum/dad than other categories, followed by time spent alone (girls: 21.9%; boys: 24.6%). Girls spent least time with brother/sister (13.9%), while boys spent least time with other grown-ups (14.1%). Boys recorded the most MVPA when with their friends or mum/dad (both 25.0%), while girls recorded the most MVPA when with their mum/dad (23.9%).

**Table 2:** Girls' after school time and MVPA by who they were with and indoor or outdoor location

		**On own**	**Friend**	**Brother/sister**	**Mum/dad**	**Other grown-up**
**Indoors**						
	Time spent (minutes)	31.3 (32.9)	20.4 (32.2)	19.9 (30.4)	42.1 (42.3)	27.8 (42.8)
	Proportion of time indoors (%)	22.1	14.4	14.1	29.8	19.6
	Proportion of all matched time (%)	20.1	13.1	12.8	27.1	17.9
	Indoor MVPA (minutes)	2.1 (3.0)	2.0 (3.8)	1.2 (2.0)	2.6 (3.0)	2.4 (4.5)
	Proportion of indoor MVPA (%)	20.4	19.4	11.7	25.2	23.3
	Proportion of all matched MVPA (%)	15.7	14.9	9.0	19.4	17.9
**Outdoors**						
	Time spent (minutes)	2.7 (8.5)	4.5 (13.1)	1.7 (7.7)	2.9 (5.8)	2.2 (6.0)
	Proportion of time outdoors (%)	19.3	32.1	12.1	20.7	15.7
	Proportion of all matched time (%)	1.7	2.9	1.1	1.9	1.4
	Outdoor MVPA (minutes)	0.5 (1.4)	1.0 (3.4)	0.4 (2.1)	0.6 (1.6)	0.6 (1.9)
	Proportion of outdoor MVPA (%)	16.1	32.2	12.9	19.4	19.4
	Proportion of all matched MVPA (%)	3.7	7.5	3.0	4.5	4.5
**Total**						
	Time spent (minutes)	34.0 (35.5)	24.9 (37.7)	21.6 (32.9)	45.0 (44.2)	30.0 (44.7)
	Proportion of all matched time (%)	21.9	16.0	13.9	28.9	19.3
	Total MVPA (minutes)	2.6 (3.7)	3.0 (6.0)	1.6 (3.3)	3.2 (3.8)	3.0 (5.1)
	Proportion of matched MVPA (%)	19.4	22.4	11.9	23.9	22.4
	Proportion of time indoors (%)	92.1	81.9	92.1	93.6	92.7
	Proportion of time outdoors (%)	7.9	18.1	7.9	6.4	7.3

MVPA: Moderate to vigorous physical activity.

Values are presented as mean (SD) after school minutes or percentages as designated.

‘Matched’ refers to periods with available accelerometer, global positioning system and diary data.

**Table 3:** Boys’ after school time and MVPA by who they were with and indoor or outdoor location

		**On own**	**Friend**	**Brother/sister**	**Mum/dad**	**Other grown-up**
**Indoors**						
	Time spent (minutes)	37.2 (40.5)	21.2 (32.8)	26.1 (34.9)	41.8 (44.1)	21.3 (37.2)
	Proportion of time indoors (%)	26.3	15.0	18.4	29.5	15.1
	Proportion of all matched time (%)	23.9	13.6	16.8	26.9	13.7
	Indoor MVPA (minutes)	2.3 (3.0)	2.7 (6.2)	1.9 (3.2)	3.1 (4.0)	2.5 (4.7)
	Proportion of indoor MVPA (%)	22.3	26.2	18.4	30.1	24.3
	Proportion of all matched MVPA (%)	17.2	20.1	14.2	23.1	18.7
**Outdoors**						
	Time spent (minutes)	2.5 (6.3)	4.0 (12.3)	1.8 (6.0)	3.8 (6.8)	1.5 (6.0)
	Proportion of time outdoors (%)	17.9	28.6	12.9	27.1	10.7
	Proportion of all matched time (%)	1.6	2.6	1.2	2.4	1.0
	Outdoor MVPA (minutes)	0.5 (1.4)	1.2 (4.0)	0.3 (1.2)	0.8 (1.9)	0.3 (1.6)
	Proportion of outdoor MVPA (%)	16.1	38.7	9.7	25.8	9.7
	Proportion of all matched MVPA (%)	3.7	9.0	2.2	6.0	2.2
**Total**						
	Time spent (minutes)	39.7 (41.8)	25.2 (38.7)	27.9 (37.2)	45.6 (46.6)	22.8 (38.4)
	Proportion of all matched time (%)	24.6	15.6	17.3	28.3	14.1
	Total MVPA (minutes)	2.8 (3.4)	3.9 (8.1)	2.2 (3.7)	3.9 (4.8)	2.8 (5.1)
	Proportion of matched MVPA (%)	17.9	25.0	14.1	25.0	17.9
	Proportion of time indoors (%)	93.7	84.1	93.5	91.7	93.4
	Proportion of time outdoors (%)	6.3	15.9	6.5	8.3	6.6

MVPA: Moderate to vigorous physical activity.

Values are presented as mean (SD) after school minutes or percentages as designated.

‘Matched’ refers to periods with available accelerometer, global positioning system and diary data.

### Time spent in different contexts

The greatest share of time outdoors was spent with friends (girls: 32.1%; boys: 28.6%), followed by mum/dad (girls: 20.7%; boys: 27.1%). Both girls (2.9%) and boys (2.6%) spent a small percentage of the total after school period outdoors with friends. Amongst girls, the smallest proportion of time outdoors was spent with brother/sister (12.1%); while for boys least time outdoors was spent with other grown-ups (10.7%). Children’s time indoors was mostly spent with mum/dad (girls: 29.8%; boys: 29.5%) or by themselves (girls: 22.1%; boys: 26.3%). Only 14.4% of girls’ and 15.0% of boys’ indoor time was spent with friends.

### MVPA recorded in different contexts

Both girls (32.2%) and boys (38.7%) most commonly recorded outdoor MVPA in the presence of friends. Least outdoor MVPA was recorded with brother/sister (girls: 12.9%; boys 9.7%) or other grown-ups for boys (9.7%). Indoor MVPA was more evenly distributed, although for both girls (25.2%) and boys (30.1%) this was most commonly recorded with mum/dad.

### Associations between time in specific contexts and after school MVPA

Table 4 (girls) and Table 5 (boys) contain data from multiple linear regression models examining relationships between hours spent in specific contexts and minutes of after school MVPA. The models explained 34.4% of girls’ and 30.1% of boys’ variance in after school MVPA. For both girls and boys, outdoor contexts exhibited stronger associations with MPVA than indoor contexts. Time spent outdoors in the company of friends was particularly important for both boys and girls, with an increase of approximately 17 minutes of MVPA recorded for every additional hour spent in this context. Similarly, when girls spent time outdoors with siblings, they recorded on average 21.21 minutes of MVPA each hour. This relationship was similar for boys but non-significant. When indoors, time with friends was positively associated with MVPA, however relationships were weaker than when outdoors (4.61 and 7.42 minute increase in MVPA accrued per hour for girls and boys respectively). Relationships of similar direction and magnitude to these were also observed between time indoors with other grown-ups and MVPA for both girls and boys. Time spent alone either indoors or outdoors was not associated with MVPA regardless of gender.

**Table 4:** Multiple linear regression of time in specific contexts and total after school MVPA amongst girls (n = 230)

		**Beta**	**95% CI**		**t**	**p**
Outdoors	On own	7.27	−1.08	15.61	1.72	0.088
	Friend	17.35	4.47	30.24	2.65	0.009
	Brother/sister	21.21	14.17	28.25	5.94	0.000
	Mum/dad	5.55	−6.34	17.45	0.92	0.359
	Other grown-up	12.76	−1.96	27.50	1.71	0.089
Indoors	On own	1.78	−0.58	4.14	1.49	0.138
	Friend	4.61	1.37	7.85	2.81	0.005
	Brother/sister	2.93	0.65	5.22	2.53	0.012
	Mum/dad	2.87	0.98	4.76	2.99	0.003
	Other grown-up	5.33	2.95	7.71	4.41	0.000
						R^2^ = 0.344

MVPA: Moderate to vigorous physical activity.

Adjusted for standardised body mass index, age, index of multiple deprivation, daylight.

Beta: mean increase in minutes of MVPA for each hour spent in that context.

**Table 5:** Multiple linear regression of time in specific contexts and total after school MVPA amongst boys (n = 197)

		**Beta**	**95% CI**		**t**	**p**
Outdoors	On own	−0.41	−13.27	12.45	−0.06	0.949
	Friend	17.53	2.76	32.31	2.34	0.020
	Brother/sister	16.95	−12.12	46.01	1.15	0.251
	Mum/dad	9.00	−6.25	24.25	1.16	0.246
	Other grown-up	8.54	−10.79	27.87	0.87	0.385
Indoors	On own	−0.64	−2.92	1.63	−0.56	0.579
	Friend	7.42	2.99	11.85	3.30	0.001
	Brother/sister	2.80	−0.14	5.74	1.88	0.062
	Mum/dad	1.77	−0.39	3.93	1.62	0.108
	Other grown-up	4.44	1.98	6.90	3.56	0.000
						R^2^ = 0.301

MVPA: Moderate to vigorous physical activity.

Adjusted for standardised body mass index, age, index of multiple deprivation, daylight.

Beta: mean increase in minutes of MVPA for each hour spent in that context.

## Discussion

The main findings of this study are that who children spend time with after school is an important influence on physical activity, and that in particular, time spent outdoors with other children is a key context for participation in MVPA. Previous studies have investigated children’s independent mobility and independent physical activity, demonstrating that greater license to leave the home unaccompanied is positively associated with time outdoors [27] and physical activity [25]. This work builds upon those findings by quantifying the time children spend alone, with adults, or with other children, and matching this with objective measures of physical activity and indoor/outdoor location. Participants reported spending most time alone or with their parents, especially during indoor time which was very rarely spent with other children. Although children spent few minutes outdoors after school, when they were outdoors they were most likely to be with friends. The accumulation of long periods spent indoors alone or supervised by adults and comparatively little time spent outdoors with other children supports the view that there are limited opportunities for primary school children to go outdoors without an adult [24,44]. This is concerning given that independent mobility has an established association with children’s physical activity [25-27], and that time outdoors is approximately three times more likely to spent engaging in MVPA [17].

It has been reported in other UK-based studies that approximately one third of children are only allowed outdoors without an adult when in the company of other children [15]. Previous work also suggests that neighbourhood relations and friends are linked to perceptions of safety for both parents and children [45,46]. Neighbourhood relations and having someone to play with may also positively influence parental decisions about independent mobility [26]. However, from the present cross sectional data it is not possible to distinguish whether time outdoors facilitates being with friends, or whether the companionship of other children is a pre-requisite of parents’ willingness to grant independent mobility. Parents may be vulnerable to a cycle of increased safety concerns linked to limited independent mobility and the subsequent social norm of children not being allowed out to play in the local environment [47,48]. Valentine & McKendrick [24] suggest that a move from public play to organised forms of physical activity has prompted suspicion of those children in public space without adults. Similarly, Ergler, Kearns & Witten [48] report that the normalisation of indoor play is especially pronounced in urban areas because children are unable to use informal areas such as sidewalks. The regression analyses report that alongside outdoor time with friends, indoor time with friends was also positively associated with MVPA. Time spent with other children therefore appears crucial for physical activity, and this is augmented by being outdoors. Recently published longitudinal data report that an increase in the number of friends between primary and secondary school is associated with an increase in girls MVPA [49]. Further longitudinal work is necessary to understand whether the formation of friendship groups is a product of, or fundamental determinant for independent mobility and outdoor physical activity. Based on such work it may be possible to promote physical activity by developing neighbourhood community links amongst children and parents, and seeking to restore the social norm of children using the outdoors as a setting for physical activity.

Parents are reported to be more protective of girls due to greater perceived risk and to subsequently limit their independence [21,22,50]. This paper supports this position indicating that indoor contexts are more important for girls’ physical activity than for boys’. Time spent indoors with friends was important for both genders, however periods indoors with siblings or parents were only associated with MVPA amongst girls. These findings echo qualitative work by Brockman, Fox & Jago [51] which reported that girls were more likely to report active play centred on the home and with family members. Previous research has reported that similar numbers of boys and girls are allowed outdoors without an adult, but that for girls this was more likely to be conditional on other children being present [15]. The strength of association between time spent outdoors with friends or siblings and MVPA in this study supports the hypothesis that girls who do have other children to accompany them outdoors are likely to be more active. Thus while safety in numbers and fostering friendship groups may be important to facilitate after school MVPA [49], it is encouraging that despite their limited independence girls appear to find ways to be active indoors. These findings tie with those of Atkin, Gorely, Biddle et al. [14] who found that technology based sedentary behaviour during the ‘critical hours’ was higher amongst boys than girls. Future research and interventions may benefit from not only increasing the time children spend outdoors with others, but also seeking to maximise the potential of indoor environments for physical activity and limiting the time children spend alone.

It is not clear why time outdoors with friends is a particularly valuable source of MVPA. It may be due to the freedom from adult rules and structure [19,52]. Alternatively, it is possible that children’s movement patterns and behaviours vary depending on whether they are with adults or other children. It has been reported that children’s movement is more meandering when away from adults [15], and some children like to do activities (such as non-permitted behaviours) outside the view of adults [53]. This paper emphasises the importance of time spent with other children, however it should also be highlighted that many children rely on adults to supervise their activity. Strategies and policy that enable adults to supervise physical activity and encourage families to be active together may be beneficial for individuals across the lifespan. This study also suggests that time spent indoors with adults other than mum/dad is positively associated with MVPA for both boys and girls, and this may be indicative of after school supervision. It is necessary to understand more about what behaviours indoors contribute to MVPA and how these may be manipulated to increase opportunities for physical activity. For example, after school clubs offer a safe indoor environment for physical activity but opportunity for this may be limited due to the inclusion of academic and snack times [54].

### Strengths and limitations

A key strength of this study is the combination of accelerometer, GPS and diary data to describe the context of children’s physical activity. This allowed exploration of not only who children spend their time with, but whether this related to objectively measured location (indoors vs. outdoors) and physical activity. Whilst the sample size was large and was drawn from a number of different primary schools representing a large English city, the results may not be generalisable to other locations or age groups. Furthermore, given that only children who provided matched accelerometer, GPS and diary data were included, the sample may not be wholly representative of the wider population. Some included participants only provided one day of combined data which may limit the reliability of the findings, however the methodology developed and the rich context-specific nature of the physical activity data provide valuable insight into children’s leisure time behaviour and is informative for further research and interventions.

Consistent with previous studies that have combined diary and objective data, there may be errors in the children’s report of their activities and consequent MVPA classification [55]. For example, children may have recorded time spent with friends when in fact they were also under the supervision of an adult. In addition a significant proportion of time between 15:00 and 22.00 was unaccounted for due to missing diary entries reporting who the children were with. Diary records were not available for 40.5% of girls and 38.3% of boys after school time, and the proportion of missing diary data increased by hour up until bedtime. The participants were asked to record what they did after school, and as such periods where their behaviour was unstructured or intermittent may be more difficult to report [56]. This may especially be the case for children who lack the cognitive and linguistic ability to describe their behaviour [57]. Unstructured activity may be more likely to reflect low intensity physical activity so a greater proportion of this might be missing data. This is supported by the fact that missing diary data contributed disproportionally fewer minutes of MVPA. However, approximately one third of MVPA was not recalled and described by children in their diary. Examining the source of this unreported physical activity should be the subject of further research.

It is also likely that there were errors in the differentiation of physical activity location by the GPS receiver. The GPS signal can be lost when outdoors and although children were trained to turn the GPS on when leaving school it is possible that some delay may have occurred leading to loss of outdoor data. Some outdoor data may therefore erroneously be classified as indoors. The present study was cross sectional, only recorded after school weekday activities and did not adjust for clustering within schools. Longitudinal work is required to fully understand the impact of the variables explored here, particularly the influence of the companionship of other children on independent mobility and unstructured outdoor physical activity. Whilst at present it is clear that children’s outdoor time with friends represents a very small proportion of leisure time, this represents an important intervention target. This is because of the potential for change during the after school period, the greater accumulation of MVPA during time spent in this context, the additional social benefits of this type of activity [20], and the harmful effects of sedentary behaviours occurring indoors [14].

## Conclusions

This study indicates that children spend most of the after school period indoors alone or with parents and very little time outdoors playing with other children. However, that time which is spent outdoors with friends makes the greatest contribution towards outdoor MVPA. Time outdoors with other children was most strongly associated with MVPA whereas time spent alone was not associated with MVPA either indoors or outdoors. In addition to promoting active time indoors, strategies to foster neighbourhood friendship groups and remove barriers which restrict time outdoors should be investigated further and considered as components of larger multi-level interventions to promote physical activity.

## Competing interests

The authors declare that they have no competing interests.

## Authors’ contributions

MP drafted the initial manuscripts and conducted the analyses. All other authors contributed to the design of the project and the writing of the manuscript. All authors read and approved the final manuscript.

## References

[B1] Janssen I Leblanc AG Systematic review of the health benefits of physical activity and fitness in school-aged children and youth Int J Behav Nutr Phys Act 2010 7 40 20459784 10.1186/1479-5868-7-40 PMC2885312

[B2] Strong WB Malina RM Blimkie CJ Daniels SR Dishman RK Gutin B Hergenroeder AC Must A Nixon PA Pivarnik JM Rowland T Trost S Trudeau F Evidence based physical activity for school-age youth J Pediatr 2005 146 732 737 15973308 10.1016/j.jpeds.2005.01.055

[B3] Department of Health Start active, stay active: a report on physical activity from the four home countries' Chief Medical Officers 2011 London: Department of Health

[B4] Davison KK Edmunds LS Wyker BA Young LM Sarfoh VS Sekhobo JP Feasibility of increasing childhood outdoor play and decreasing television viewing through a family-based intervention in WIC, New York State, 2007–2008 Prev Chronic Dis 2011 8 A54 21477494 PMC3103559

[B5] Riddoch CJ Mattocks C Deere K Saunders J Kirkby J Tilling K Leary SD Blair SN Ness AR Objective measurement of levels and patterns of physical activity Arch Dis Child 2007 92 963 969 17855437 10.1136/adc.2006.112136 PMC2083612

[B6] Sallis JF Owen N Fotheringham MJ Behavioral epidemiology: a systematic framework to classify phases of research on health promotion and disease prevention Ann Behav Med 2000 22 294 298 11253440 10.1007/BF02895665

[B7] Bauman AE Reis RS Sallis JF Wells JC Loos RJ Martin BW Correlates of physical activity: why are some people physically active and others not? Lancet 2012 380 258 271 22818938 10.1016/S0140-6736(12)60735-1

[B8] Sallis JF Owen N Physical activity and behavioral medicine 1999 Thousand Oaks, CA: Sage Publications

[B9] Giles-Corti B Timperio A Bull F Pikora T Understanding physical activity environmental correlates: Increased specificity for ecological models Exerc Sport Sci Rev 2005 33 175 181 16239834 10.1097/00003677-200510000-00005

[B10] Humpel N Owen N Leslie E Environmental factors associated with adults' participation in physical activity: a review Am J Prev Med 2002 22 188 199 11897464 10.1016/s0749-3797(01)00426-3

[B11] Page AS Cooper AR Griew P Jago R Independent mobility, perceptions of the built environment and children's participation in play, active travel and structured exercise and sport: the PEACH Project Int J Behav Nutr Phys Act 2010 7 1 10 20170504 10.1186/1479-5868-7-17 PMC2837609

[B12] Sallis JF Owen N Fisher EB Glanz K, Rimer BK, Lewis FM Ecological Models of Health Behaviour Health Behaviour and Health Education: Theory, Research and Practice 2008 San Francisco, CA: Jossey Bass 465 483

[B13] Dunton GF Liao Y Intille S Wolch J Pentz MA Physical and social contextual influences on children's leisure-time physical activity: an ecological momentary assessment study J Phys Act Health 2011 8 Suppl 1 S103 108 21350250 10.1123/jpah.8.s1.s103

[B14] Atkin AJ Gorely T Biddle SJH Marshall SJ Cameron N Critical hours: Physical activity and sedentary behavior of adolescents after school Pediatr Exerc Sci 2008 20 446 456 19168921 10.1123/pes.20.4.446

[B15] Mackett R Brown B Gong Y Kitazawa K Paskins J Children's independent movement in the local environment Built Environ 2007 33 454 468

[B16] Cleland V Crawford D Baur LA Hume C Timperio A Salmon J A prospective examination of children's time spent outdoors, objectively measured physical activity and overweight Int J Obes 2008 32 1685 1693 10.1038/ijo.2008.171 18852701

[B17] Cooper AR Page AS Wheeler BW Hillsdon M Griew P Jago R Patterns of GPS measured time outdoors after school and objective physical activity in English children: the PEACH project Int J Behav Nutr Phys Act 2010 7 31 20412582 10.1186/1479-5868-7-31 PMC2867989

[B18] Veitch J Bagley S Ball K Salmon J Where do children usually play? A qualitative study of parents' perceptions of influences on children's active free-play Health Place 2006 12 383 393 16814197 10.1016/j.healthplace.2005.02.009

[B19] Brockman R Jago R Fox KR Children's active play: self-reported motivators, barriers and facilitators BMC Publ Health 2011 11 461 10.1186/1471-2458-11-461 PMC3124432 21663605

[B20] Burdette H Whitaker R Resurrecting free play in young children Arch Pediatr Adolesc Med 2005 159 46 50 15630057 10.1001/archpedi.159.1.46

[B21] Carver A Timperio A Hesketh K Crawford D Are children and adolescents less active if parents restrict their physical activity and active transport due to perceived risk? Soc Sci Med 2010 70 1799 1805 20347200 10.1016/j.socscimed.2010.02.010

[B22] Carver A Timperio A Hesketh K Crawford D How does perceived risk mediate associations between perceived safety and parental restriction of adolescents' physical activity in their neighborhood? Int J Behav Nutr Phys Act 2012 9 57 22607169 10.1186/1479-5868-9-57 PMC3458944

[B23] Hillman M Children's rights and adults' wrongs Children's Geographies 2006 4 61 67

[B24] Valentine G McKendrick J Children's outdoor play: Exploring parental concerns about children's safety and the changing nature of childhood Geoforum 1997 28 219 235

[B25] Page AS Cooper AR Griew P Davis L Hillsdon M Independent mobility in relation to weekday and weekend physical activity in children aged 10–11 years: The PEACH Project Int J Behav Nutr Phys Act 2009 6 1 9 19128458 10.1186/1479-5868-6-2 PMC2636750

[B26] Prezza M Pilloni S Morabito C Sersante C Alparone FR Giuliani MV The influence of psychosocial and environmental factors on children's independent mobility and relationship to peer frequentation J Community Appl Soc Psychol 2001 11 435 450

[B27] Wen LM Kite J Merom D Rissel C Time spent playing outdoors after school and its relationship with independent mobility: a cross-sectional survey of children aged 10–12 years in Sydney, Australia Int J Behav Nutr Phys Act 2009 6 1 8 19291324 10.1186/1479-5868-6-15 PMC2661046

[B28] Jago R Thompson JL Page AS Brockman R Cartwright K Fox KR Licence to be active: parental concerns and 10-11-year-old children's ability to be independently physically active J Pub Health 2009 31 472 477 10.1093/pubmed/fdp053 PMC2781721 19505927

[B29] Thomson JL Philo C Playful spaces? a social geography of children's play in Livingston, Scotland Children's Geographies 2004 2 111 130

[B30] Veitch J Salmon J Ball K Individual, social and physical environmental correlates of children's active free-play: a cross-sectional study Int J Behav Nutr Phys Act 2010 7 1 10 20181061 10.1186/1479-5868-7-11 PMC2841089

[B31] O'Brien M Jones D Sloan D Rustin M Children's independent spatial mobility in the urban public realm Childhood 2000 7 257 277

[B32] Troiano RP Berrigan D Dodd KW Masse LC Tilert T McDowell M Physical activity in the United States measured by accelerometer Med Sci Sports Exerc 2008 40 181 188 18091006 10.1249/mss.0b013e31815a51b3

[B33] Toftager M Kristensen PL Oliver M Duncan S Christiansen LB Boyle E Brond JC Troelsen J Accelerometer data reduction in adolescents: effects on sample retention and bias Int J Behav Nutr Phys Act 2013 10 140 24359480 10.1186/1479-5868-10-140 PMC3880051

[B34] Rodriguez DA Brown AL Troped PJ Portable global positioning units to complement accelerometry-based physical activity monitors Med Sci Sports Exerc 2005 37 S572 581 16294120 10.1249/01.mss.0000185297.72328.ce

[B35] McKenna J Foster LJ Page AS Exploring recall of physical activity in young people using qualitative interviewing Pediatr Exerc Sci 2004 16 5 14

[B36] Page AS Cooper AR McKenna J Foster LJ Riddoch CJ Fox KR Development of a research tool to measure physical activity among young people aged 5–16 in the UK Meas Phys Educ Exerc Sci 2000 4 267 268

[B37] Cole TJ Bellizzi MC Flegal KM Dietz WH Establishing a standard definition for child overweight and obesity worldwide: international survey BMJ 2000 320 1240 1243 10797032 10.1136/bmj.320.7244.1240 PMC27365

[B38] Sunrise and sunset for UK (Bristol) [http://www.timeanddate.com/worldclock/astronomy.html?n=304]

[B39] Evenson KR Catellier DJ Gill K Ondrak KS McMurray RG Calibration of two objective measures of physical activity for children J Sports Sci 2008 26 1557 1565 18949660 10.1080/02640410802334196

[B40] Trost SG Loprinzi PD Moore R Pfeiffer KA Comparison of accelerometer cut points for predicting activity intensity in youth Med Sci Sports Exerc 2011 43 1360 1368 21131873 10.1249/MSS.0b013e318206476e

[B41] Wheeler BW Cooper AR Page AS Jago R Greenspace and children's physical activity: a GPS/GIS analysis of the PEACH project Prev Med 2010 51 148 152 20542493 10.1016/j.ypmed.2010.06.001

[B42] Maddison R Ni Mhurchu C Global positioning system: a new opportunity in physical activity measurement Int J Behav Nutr Phys Act 2009 6 73 19887012 10.1186/1479-5868-6-73 PMC2777117

[B43] Reilly JJ Penpraze V Hislop J Davies G Grant S Paton JY Objective measurement of physical activity and sedentary behaviour: review with new data Arch Dis Child 2008 93 614 619 18305072 10.1136/adc.2007.133272

[B44] Karsten L It all used to be better? Different generations on continuity and change in urban children's daily use of space Children's Geographies 2005 3 275 290

[B45] Mikkelsen MR Christensen P Is children's independent mobility really independent? A study of children's mobility combining ethnography and GPS/mobile phone technologies Mobilities 2009 4 37 58

[B46] Valentine G "Oh yes I can." "oh no you can't": Children and parents' understandings of kids' competence to negotiate public space safely Antipode 1997 29 65 89

[B47] Carver A Timperio A Crawford D Playing it safe: The influence of neighbourhood safety on children's physical activity - A review Health Place 2008 14 217 227 17662638 10.1016/j.healthplace.2007.06.004

[B48] Ergler CR Kearns RA Witten K Seasonal and locational variations in children's play: Implications for wellbeing Soc Sci Med 2013 91 178 185 23312793 10.1016/j.socscimed.2012.11.034

[B49] Jago R Page AS Cooper AR Friends and physical activity during the transition from primary to secondary school Med Sci Sports Exerc 2012 44 111 117 21697746 10.1249/MSS.0b013e318229df6e

[B50] Hillman M Adams J Whitelegg J One False Move: A Study of Children's Independent Mobility 1990 London: PSI Publishing

[B51] Brockman R Fox KR Jago R What is the meaning and nature of active play for today's children in the UK? Int J Behav Nutr Phys Act 2011 8 15 21385336 10.1186/1479-5868-8-15 PMC3059273

[B52] Veitch J Salmon J Ball K Children's perceptions of the use of public open spaces for active free-play Children's Geographies 2007 5 409 422

[B53] Soori H Bhopal RS Parental permission for children's independent outdoor activities. Implications for injury prevention Eur J Public Health 2002 12 104 109 12073747 10.1093/eurpub/12.2.104

[B54] Trost SG Rosenkranz RR Dzewaltowski D Physical activity levels among children attending after-school programs Med Sci Sports Exerc 2008 40 622 629 18317385 10.1249/MSS.0b013e318161eaa5

[B55] Goodman A Mackett RL Paskins J Activity compensation and activity synergy in British 8–13 year olds Prev Med 2011 53 293 298 21820009 10.1016/j.ypmed.2011.07.019

[B56] Kohl HW Fulton JE Caspersen CJ Assessment of physical activity among children and adolescents: A review and synthesis Prev Med 2000 31 S54 S76

[B57] Trost SG Morgan AM Saunders R Felton G Ward DS Pate RR Children's understanding of the concept of physical activity Pediatr Exerc Sci 2000 12 293 299

